# Clinical Manifestations of Herpes Zoster Associated with Complications in Children

**DOI:** 10.3390/children8100845

**Published:** 2021-09-24

**Authors:** Dong Ha Kang, Byung Ok Kwak, A Young Park, Han Wool Kim

**Affiliations:** 1 Department of Pediatrics, Hallym University Sacred Heart Hospital, Anyang 14068, Korea; eastdown@hallym.or.kr (D.H.K.); aypark12@hallym.or.kr (A.Y.P.); 2 Department of Pediatrics, Hallym University Kangnam Sacred Heart Hospital, Seoul 07441, Korea; qquack00@hallym.or.kr

**Keywords:** herpes zoster, children, varicella-zoster virus

## Abstract

Herpes zoster (HZ) is caused by latent varicella-zoster virus (VZV) reactivation when VZV-specific cell-mediated immunity declines. Information on HZ in children is limited. Therefore, we retrospectively investigated HZ’s clinical course and complications in children. We extracted the outpatient and hospitalization medical records of pediatric patients (<19 years) primarily diagnosed with HZ (ICD-10 B02 code) between January 2010 and November 2020. HZ was defined as a typical unilateral dermatomal vesicular rash where HZ was the treating physician’s primary diagnosis. Recognized HZ complications included combined bacterial skin infection, ophthalmic zoster, zoster oticus without facial paralysis, meningitis, and PHN. We identified 602 HZ cases, among which 54 developed HZ complications and were included in our analysis. The median age was 14.7 years, most patients were aged ≥13 years (42, 79%), and none were aged <4 years. Fifty-three were immunocompetent, and only one had systemic lupus erythematosus. The most frequent complication was zoster ophthalmicus (*n* = 26, 48%). HZ complications were also observed in immunocompetent or vaccinated children exhibiting a head or neck rash before and after VZV immunization. Current VZV vaccination programs may be insufficient in preventing HZ complications. Therefore, close varicella and HZ burden monitoring and the establishment of effective VZV vaccination programs are imperative.

## 1. Introduction

Herpes zoster (HZ) is caused by the reactivation of the latent varicella-zoster virus (VZV) when VZV-specific cell-mediated immunity declines, and it is characterized by small blisters and pain in the corresponding unilateral skin segment [1]. The incidence of HZ has been estimated at 3–5 cases per 1000 person-years worldwide, and it increases with age [2]. In recent decades, temporal increases in HZ incidence have been reported globally [3]. The vaccination strategy could affect HZ epidemiology, as childhood varicella vaccination potentially increases HZ incidence in the adult population by decreasing exogenous boosting [4]. Another possible reason underlying the increasing incidence, similar to that for wild-type VZV, is the reactivation of the latent infection established by vaccination, resulting in HZ. In vaccinated children, HZ is potentially caused by vaccine-strain or wild-type VZV acquired either from unrecognized infection before or after vaccination or breakthrough varicella [5,6,7,8,9,10]. In South Korea, a single-dose varicella vaccine was included in the National Immunization Program (NIP) for 12–15-month-old children in 2005, and the varicella vaccine coverage was estimated at over 95% in children born after 2007 [11]. The live, attenuated vaccine against HZ for older adults was introduced in 2012 [12]. These immunization policies might have influenced HZ incidence in Korea and were indeed shown to increase the incidence [13]. 

HZ causes several complications, including postherpetic neuralgia (PHN), bacterial skin infection, ocular complication, motor neuropathy, and meningitis. In addition to disease incidence, these complications are also an important consideration in the establishment of vaccination policy. Although HZ incidence in immunocompetent children had initially been considered to be low and mild with minimal pain [14], recent reports have also demonstrated a relatively higher incidence in such children than that in the past [15,16]. In addition, the occurrence of complications involving functionally important organs, such as zoster oticus and ophthalmicus, potentially has a significant impact on the development of children and adolescents; therefore, it is important to monitor HZ incidence and complications in order to establish vaccination policies that help prevent HZ. HZ complications in older people have been well investigated; however, information regarding HZ in children has been relatively scarce. Therefore, we retrospectively reviewed the clinical course and complications of HZ in children.

## 2. Materials and Methods

### 2.1. Study Design and Population

This retrospective, observational study was conducted at Hallym University Sacred Heart Hospital and Kangnam Sacred Heart Hospital in South Korea. We extracted all the medical records regarding the outpatient visits and hospitalizations of pediatric patients (aged < 19 years) with HZ (ICD-10 B02 code) as the primary diagnosis between January 2010 and November 2020. An HZ case was defined as a patient with a typical unilateral dermatomal vesicular rash in which HZ was the treating physician’s primary diagnosis, when the patient was managed in a manner consistent with HZ (by medication or observation), and when no alternative diagnoses emerged from diagnostic testing or subsequent events. Among patients with HZ, those who had experienced complications were enrolled. In this study, the following diseases were recognized as HZ complications: combined bacterial skin infection, ophthalmic zoster, zoster oticus without facial paralysis, meningitis, and PHN. Combined bacterial infection was identified using medical records or microbiological results, and ophthalmic zoster was diagnosed as keratitis, retinitis, conjunctivitis, or uveitis by an ophthalmologist. Zoster oticus without facial paralysis was defined as inner- or middle-ear inflammation examined by an otorhinolaryngologist. Meningitis was confirmed by analysis of cerebrospinal fluid for VCV DNA using the polymerase chain reaction test. PHN was defined as the prescription of pain medication for at least 2 months after rash onset. This study was approved by the Institutional Review Board of Hallym University Sacred Heart Hospital (IRB No. 2021-06-021), and the ethic approved date 21 July 2021.

### 2.2. Data Collection Analysis

Demographic data regarding age, sex, history of varicella and VZV vaccination, and underlying diseases were extracted. The collected clinical information included that regarding dermatome, antiviral prescriptions, treatment duration, and hospitalization. 

Data was entered in Microsoft Excel^®^ 2013 (Microsoft, Redmond, MA, USA). Descriptive analysis for categorical and continuous variables was performed. Categorical variable results were expressed as frequency (percentages), and continuous variable results were expressed as mean and standard deviation (SD) or median and range, depending on the variable’s distribution.

## 3. Results

**Figure 1:**
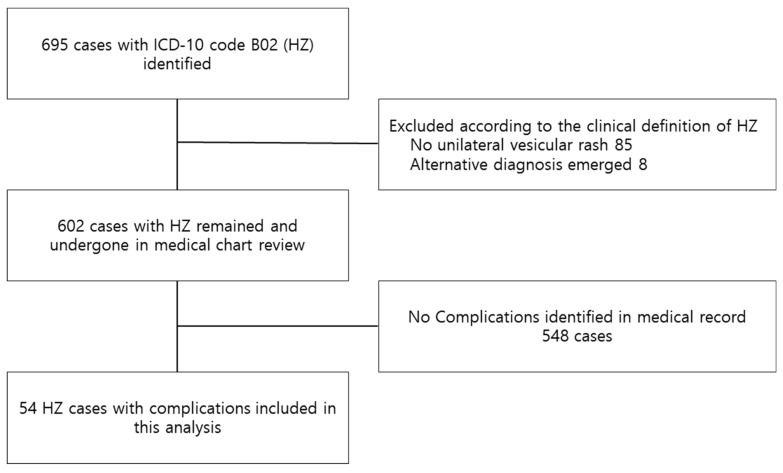
Flow chart of the patient-selection process. HZ, Herpes zoster.

**Figure 2:**
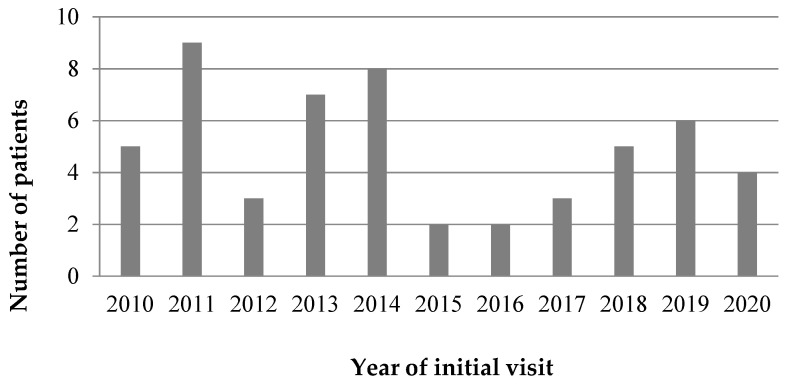
Distribution of the number of cases according to year of initial visit.

**Figure 3:**
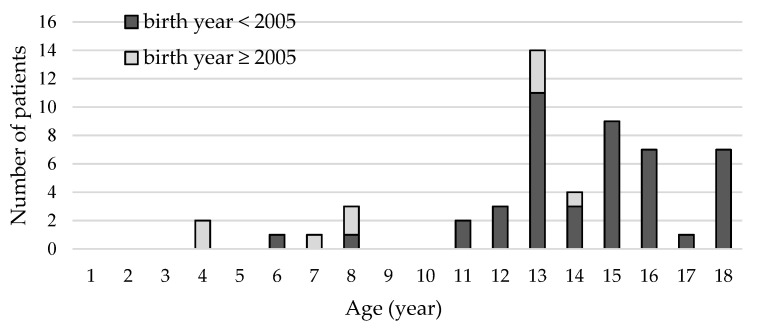
Distribution of age at onset of herpes zoster with complications.

A total of 602 HZ cases were identified during the study period (Figure 1). Among these, 54 cases developed HZ complications and were included in our analysis. Patient demographic is shown in Table 1. The median age was 14.7 years, and the male-to-female ratio was 1.6:1. Figure 2 shows the distribution of the number of complicated herpes zoster cases according to year of initial visit. The age distribution at diagnosis is shown in Figure 3. Most patients were 13 years of age or older (42, 78%), and none were younger than 4 years of age. Patients born before 2005 predominantly developed HZ after the age of 10; however, those born after 2005, that is, after the introduction of the NIP, were evenly distributed across all age groups (Figure 3). Fifty-three (98%) were immunocompetent, and only one (2%) had systemic lupus erythematosus and was under hydroxychloroquine treatment. Among seven patients, whose past varicella infection history was recorded, four had previous varicella infection. Two of the three patients who had reportedly never experienced varicella had been vaccinated for VZV in the past. Ten patients had a confirmed varicella-immunization history. The VZV vaccination history of the remaining 44 patients could not be located in their medical records, although three of them were born after the introduction of the VZV vaccine in the NIP (2005).

**Figure 4:**
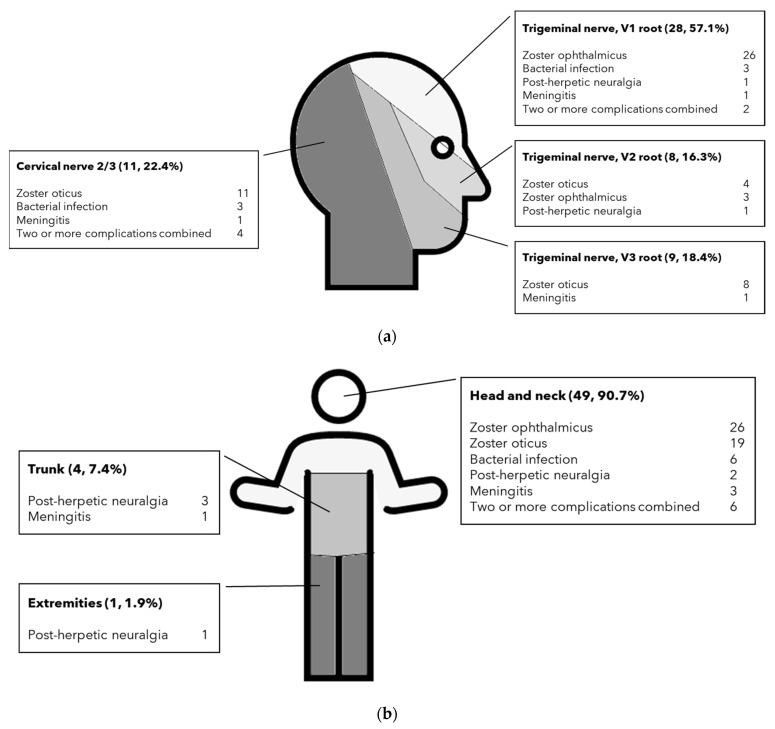
Distribution of complications according to dermatome: (**a**) head and neck involvement (**b**) whole-body distribution.

The most common site of the rash was the head or neck, followed by the trunk and extremities (Figure 4). In cases of trigeminal nerve root involvement, the ophthalmic branch was frequently involved, and almost all complications were zoster ophthalmicus cases. The proportions of HZ-complication categories are shown in Table 2. The most frequent complication was zoster ophthalmicus. Among the zoster ophthalmicus complications, conjunctivitis was the most common (*n* = 25), followed by keratitis, uveitis, and glaucoma (*n* = 10, 2, and 1, respectively). Zoster oticus was the second most frequent complication. Ramsay Hunt syndrome (zoster oticus with facial paralysis) accounted for 57.9% of ear involvement cases, and these patients completely recovered.

Neurological manifestations, including facial paralysis and meningitis, were detected in 15 cases (27%). Regarding meningitis, all four patients initially complained of headache, with the vesicular rash subsequently developing 1–5 days later. VZV DNA was detected in their cerebrospinal fluid. Three of them exhibited skin lesions on their faces, and one patient developed the rash on his trunk (T12 dermatome). All of them were admitted to the hospital, where they intravenously received antiviral agents. 

Six patients had combined bacterial skin infection, and four exhibited cellulitis on the involved skin area. Six patients developed PHN, involving various nerve roots. Facial and thoracic nerve roots were involved in two cases, and the other cases involved the lumbar and cervical roots. Their mean age was 16.1 years (range: 13.5–18.5). All patients with PNH were treated with pregabalin or gabapentin. Additional tricyclic antidepressants were given to 2 patients (each patient with amitriptyline or nortriptyline), and 2 patients were injected with intrathecal glucocorticoid. No opioids were given to any patients.

Thirty cases (56%) exclusively received acyclovir, 11 (20%) received famciclovir only, and four (7%) received valacyclovir during the treatment period. The treatment period ranged from five to 14 days, with a median of 7 days (interquartile range: 5.0–7.0). Intravenous antiviral administration was used in 25 cases (46%), and 29 cases (54%) were hospitalized. 

Most patients completely recovered, and all HZ oticus patients were fully cured without hearing loss. All except one patient with HZ ophthalmicus recovered without any sequelae. In one case with uveitis, corneal opacity was observed over 15 months, and the patient was subsequently transferred to another hospital. In three patients with PHN, complete pain relief was observed within 5 months of follow-up at the outpatient clinic. Pain persisted for 4 and 8 months in two patients with PHN, and they were subsequently lost to follow-up at the outpatient clinic. One immunocompromised patient diagnosed with systematic lupus erythematosus (SLE) experienced pain for more than 10 months and continued taking pain relief medications.

## 4. Discussion

This study investigated HZ complications in children. The results indicated that HZ complications might also occur in immunocompetent and vaccinated young children. Neurological manifestations and the involvement of sensory organs were major complications in this observation. The most common lesion locations were the head and neck areas. Hospitalization or intravenous antiviral treatment was required in many patients.

The increasing incidence of HZ is a global phenomenon, and it has been reported in Europe, the United States, and Asia [2,3,17,18]. Based on the Hope–Simpson hypothesis, which postulated that immunity against VZV reactivation in adults could be exogenously boosted by exposure to circulating varicella [19], a decline in varicella through the universal VZV vaccination program in children potentially leads to an unintended temporary increase in HZ incidence [20]. In South Korea, the VZV vaccine was introduced in 1988, and its use has become more widespread since the mid-1990s. In 2005, a single-dose vaccine targeting children aged 12–15 months was included in the NIP. Vaccine coverage was approximately 73% in 2005 before the NIP was established; by 2011, it had increased to >97% [11]. Indeed, a recent report found that as the varicella vaccination program matured, the incidence of varicella decreased by 67.5%; nonetheless, the incidence of HZ increased [13,21]. The waning of the effectiveness of the VZV vaccine has been shown in a nationwide population cohort study, which may be one of the possible reasons for the partial reduction in incidence [22]. However, these changes differed by age group, and HZ incidence was relatively low for children [13]. Our results also found that a relatively lower number of cases were observed in younger children than those in the older age group and that the total number of cases tended to decrease over time.

HZ complications, particularly persistent PHN, and hospitalization have commonly been observed in older or immunocompromised patients. In a systematic review of HZ complications, the risk of PHN varied from 5% to more than 30% [2]. In Israel, a population-based study found the proportions of PHN among adults aged 55 years and over with HZ to be 10.5–18.5% and demonstrated an increasing rate according to age [23]. In a large cohort study among adults in the United Kingdom, the estimated risks of PHN and other complications were higher in older adults [24]. However, although our study focused on children, 9.0% of the HZ cases were complicated cases. Among them, PHN accounted for 11%, and hospitalization was required in 54%. Most patients did not have any underlying diseases. Previous studies on children have also demonstrated that complicated cases tend to develop in immunocompetent children [25,26], and German data showed that immunocompetent children accounted for most hospitalizations and complications (25). Therefore, awareness regarding HZ complications and preventive measures is not only important in older people but also in immunocompetent and younger populations.

Among HZ complications, zoster ophthalmicus was the most frequent in our study. Zoster ophthalmicus develops in 10.1–14.9% of patients with HZ, and it is regarded as the most common complication besides PHN in adults with HZ [2,23]. Ocular involvement in HZ was also the second most common complication after bacterial skin infection in a previous study on children [26]. It is an important complication because zoster ophthalmicus has resulted in visual loss in 6% of cases [2]. One case of uveitis in our study did not completely recover. 

PHN is regarded the most common complication in adults and an important complication of HZ due to its impact on quality of life. However, the incidence in children has been low, as in our study [25,26]. In this analysis, the involved nerve roots varied from cranial to lumbar, and PHN cases predominantly involved older aged patients. One patient with SLE who was receiving immunosuppressants had suffered from PHN for more than 10 months.

Meningitis, another complication of HZ, is not very common in immunocompetent pediatric patients [27]. However, in this analysis, there were four cases, and their HZ lesions were primarily located on the head and neck. All of them were immunocompetent, and they recovered without any sequelae; nonetheless, hospitalization and intravenous antiviral treatment were required.

Vaccination policies against varicella vary from country to country due to concerns regarding universal varicella-vaccination’s potential to increase HZ incidence by reducing exogenous boosting to natural varicella or causing the disease burden to shift towards older individuals at higher risk of severe varicella [4]. In the United States, Canada, and Germany, a two-dose varicella vaccine schedule has been implemented. However, the single-dose regimen was adopted in the Korean NIP. In a recent Korean study, the authors revealed an increased HZ incidence and several varicella cases despite the high vaccine coverage [28]. They suggested that a second-dose varicella vaccine should be included in the NIP to reduce breakthrough varicella, and vaccination against HZ should be more actively encouraged. In this analysis, we also found that complicated HZ cases developed in many immunocompetent children. Using mathematical modelling, Suh et al. demonstrated in a recent study that two-dose VZV vaccination might significantly reduce HZ incidence [29]. Therefore, preventive measures for reducing these complications should be considered, including modification of the current VZV vaccination policy in Korea.

Notwithstanding, this study has certain limitations. First, it was conducted at two hospitals that do not entirely cover the community; hence, its generalizability is limited. Therefore, the incidence rate could not be calculated and compared to that in other population-based studies. In particular, our hospitals are equipped with an intensive care unit that treats critically ill patients. It is likely that the proportion of HZ complications in the general pediatric population is lower. Further, the distribution of complication types might have also been skewed. Second, the number of patients was not large enough to conduct robust statistical analyses. Finally, as this was a retrospective review based on medical records, the analysis was limited by possibly inaccurate descriptions of rash locations and omission of immunization or varicella history. 

## 5. Conclusions

In conclusion, HZ complications were frequently observed, even in immunocompetent or vaccinated children, and they were characterized by a rash on the head or neck before and after the introduction of VZV immunization. Current VZV vaccination programs may be not sufficient to prevent HZ complications. Therefore, close monitoring of the varicella and HZ burden and the establishment of an effective VZV vaccination program are warranted.

## Figures and Tables

**Table 1:** Demographic characteristics of patients.

Variables	*n* = 54
Age, years	
Median (range)	14.7 (4.1–18.7)
Mean (SD)	14.0 (±3.4)
Sex (female)	
*n*, (%)	21 (39)
Underlying diseases *, *n* (%)	1 (2)
History of varicella, *n* (%)	
Yes	4 (7)
No	3 (6)
Unknown	47 (87)
VZV vaccination, *n* (%)	
Yes	10 (19)
No	0 (0)
Unknown **	44 (81)

SD, Standard deviation; VZV, Varicella-zoster virus. * Systematic lupus erythematosus (*n* = 1); clinical remission state under hydroxychloroquine treatment. ** Three patients were born after introduction of the varicella-zoster vaccine in the National Immunization Program (2005).

**Table 2:** Types of complications (*n* = 54).

Complications	*n* (%)
Zoster ophthalmicus	26 (48)
Zoster oticus with facial paralysis	11 (20)
Zoster oticus	8 (15)
Postherpetic neuralgia	6 (11)
Combined bacterial infection	6 (11)
Meningitis	4 (7)
More than one complication *	6 (11)

* Combined bacterial skin infection with zoster oticus or zoster ophthalmicus; Meningitis with zoster oticus.

## Data Availability

The datasets generated and analyzed during the current study are not publicly available due to privacy and ethical concerns but are available from the corresponding author on reasonable request.
